# Myxedema Coma and Acute Hepatopathy in a Dog with Severe Atherosclerosis

**DOI:** 10.1155/2021/6622767

**Published:** 2021-10-29

**Authors:** Leah C. Giralico, Rebecca Makii, Betsy A. Pray, Valerie J. Parker

**Affiliations:** ^1^Department of Veterinary Clinical Sciences, The Ohio State University College of Veterinary Medicine, USA; ^2^Department of Veterinary Biosciences, The Ohio State University College of Veterinary Medicine, USA

## Abstract

A 9-year-old male intact mixed-breed dog was presented to The Ohio State University Veterinary Medical Center for evaluation of two days' duration of weakness, lethargy, inappetence, and one episode of vomiting the day of presentation. On presentation, the dog was depressed and tetraparetic. He was noted to be icteric and dehydrated. Obesity and truncal alopecia with a “rat tail” appearance were observed. Diagnostic testing revealed evidence of an acute hepatopathy and peritonitis. Given the dog's neurologic status, physical examination abnormalities, including a “tragic facial expression”, and hyperlipidemia, there was concern for possible myxedema coma. A thyroid panel was consistent with hypothyroidism. The dog experienced respiratory arrest prior to initiation of therapy, and an autopsy confirmed the presence of subacute necrotizing cholangiohepatitis, marked atherosclerosis, and severe thyroid atrophy. These clinical and pathologic changes were supportive of myxedema coma.

## 1. Introduction

Acute liver injury is an important cause of morbidity in dogs. There are many possible causes of acute liver injury and subsequent liver failure, with hepatotoxicity, infection, inflammatory disease, trauma, and hypoxemia accounting for most presentations [1]. Treatment involves supportive care in the form of fluid resuscitation, nutritional and electrolyte management, and hepatoprotective medications. Addressing the underlying cause, when identifiable, can also help treat acute hepatopathies [1].

Acquired primary hypothyroidism most commonly results either from immune-mediated lymphocytic thyroiditis or idiopathic atrophy; the latter is often presumed to represent the end-stage of the former. The disease is characterized by a deficiency of the thyroid hormones thyroxine (T4) and triiodothyronine (T3). The most common clinical signs include lethargy, weight gain, endocrine alopecia, and other dermatologic conditions [2, 3]. Nervous involvement in hypothyroidism is rare, but the disease has been associated with peripheral neuropathy and paresis [4].

Myxedema coma is a rare syndrome that can be seen with severe hypothyroidism. In addition to the typical signs of hypothyroidism, dogs with myxedema coma may present with dullness, myxedema (nonpitting edema of the skin), hypothermia, bradycardia, hypotension, and hypoventilation. This can progress to stupor and coma.

## 2. Case Presentation

### 2.1. History

A 9-year-old male intact mixed-breed dog was presented to The Ohio State University Veterinary Medical Center for evaluation of two days' duration of weakness, lethargy, inappetence, and one episode of vomiting the day of presentation. Seven months prior to evaluation, the dog was evaluated by an emergency clinic for acute vomiting and lethargy. Diagnostic evaluation was declined by the owner at that visit, and the dog received subcutaneous fluids and an antiemetic (maropitant, 1 mg/kg SQ once).

### 2.2. Physical Exam and Preliminary Investigation

Upon presentation to The Ohio State University Veterinary Medical Center (OSU-VMC), the dog weighed 25.6 kg and had a body condition score of 8/9 (obesity) with normal muscle condition. The dog was estimated to be approximately 7-8% dehydrated, and the mucus membranes were tacky, pale pink, and icteric. Temperature (*T*—100.0 F) and heart rate (102 beats per minute) were within normal limits. There was mild tachypnea (42 breaths per minute) with referred upper airway sounds. Heart and lung sounds were normal. His abdomen was mildly distended and tense on palpation. Rectal examination was unremarkable. Hypotrichosis of the trunk and at the tail base (rat tail) was noted. Testicular asymmetry was identified. The dog was assessed by the OSU-VMC Neurology Service to be depressed and tetraparetic with conscious proprioceptive deficits in all limbs.

Initial diagnostics performed included a packed cell volume/total protein (PCV/TP), venous blood gas, and blood pressure measurement. The blood pressure via oscillometric measurement was 125/70 mmHg (systolic/diastolic). The PCV/TP were 40% and 11 g/dL, respectively. The blood gas revealed a mild hyponatremia (138.4 mmol/L; reference range, 143.0-150.0 mmol/L), hypochloremia (106.4 mmol/L; reference range, 111.0-119.0 mmol/L), and hyperlactatemia (5.5 mmol/L; reference range, 0.5-3.5 mmol/L). BUN and creatinine at presentation were 21 and 1.5 mg/dL, respectively.

The dog was admitted to the hospital for further diagnostic evaluation and supportive care including intravenous fluids (Plasmalyte), to correct for dehydration, as well as an antiemetic (ondansetron, 0.3 mg/kg IV q8h).

The CBC (Table 1) revealed a moderate microcytic nonregenerative anemia (HCT -33%; reference range, 40-59%). A mild leukocytosis (23.7 × 10^3^/*μ*L; reference range, 4.8-13.9 × 10^3^/*μ*L) was characterized by a neutrophilia (20.6 × 10^3^/*μ*L; reference range, 2.6-10.8 × 10^3^/*μ*L) with a left shift (bands −2.6 × 10^3^/*μ*L; reference range, 0-0.1 × 10^3^/*μ*L). Serum biochemistry (Table 2) revealed a moderate mixed hepatocellular-cholestatic hepatopathy: ALT (4,563 IU/L; reference range, 18-108 IU/L); AST (1,585 IU/L; reference range, 16-51 IU/L); ALP (2,341 IU/L; reference range, 12-133 IU/L); and total bilirubin (4.24 mg/dL; reference range, 0.05-0.2 mg/dL). Also noted was moderate hyperlipidemia: cholesterol (1,272 mg/dL; reference range, 122-345 mg/dL) and triglycerides (1,118 mg/dL; reference range, 34-265 mg/dL).

Urine was obtained via cystocentesis and submitted for urinalysis. Urine specific gravity was 1.011 (after dog had received IV fluids for at least four hours). Hematuria (blood 3+), proteinuria (3+), and bilirubinuria (2+) were noted.

Both prothrombin time (PT) and partial thromboplastin time (PTT) were increased above reference intervals (20.7 seconds; reference range, 14-19 seconds and >200 seconds; reference range, 75-105 seconds, respectively). A point-of-care immunoglobulin detection test was negative for leptospirosis.

### 2.3. Diagnostic Imaging

Thoracic radiographs revealed a diffuse unstructured interstitial pattern consistent with pulmonary hypoinflation. Mineralization of the principal bronchi and multiple pinpoint mineral foci throughout the lungs were observed. The cardiac silhouette was unremarkable, and there was no evidence of pulmonary neoplasia.

**Figure 1:**
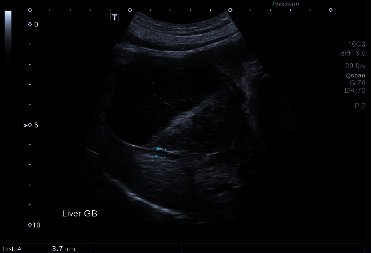
Ultrasonographic image of the gallbladder, demonstrating a thickened and hypoechoic wall, consistent with edema. The surrounding fat is hyperechoic.

Abdominal ultrasound revealed hepatomegaly; the liver was diffusely hyperechoic with a fine echotexture. The gallbladder contained a large amount of echogenic material. The gallbladder wall was thickened and hypoechoic, consistent with edema, and the fat surrounding the gallbladder was hyperechoic (Figure 1). Both the common bile and cystic ducts were distended, measuring up to 6.9 mm and 5.5 mm, respectively. The pancreas appeared normal. Gastric atony was suspected based on a fluid-filled stomach and lack of motility during examination.

There was a scant amount of anechoic to mildly echogenic peritoneal effusion present. Abdominocentesis was performed, and fluid analysis was consistent with an exudative process (white blood cells 69,000 IU/L: 85% neutrophils (degenerative neutrophils present), 15% large mononuclear cells; total protein 8 g/dL). Abdominal fluid glucose and lactate (69 mg/dL and 5.1 mmol/L) were compared to peripheral blood glucose and lactate (76 mg/dL and 3.6 mmol/L); results were not consistent with abdominal sepsis. Aerobic and anaerobic bacterial cultures of the peritoneal fluid were ultimately negative.

### 2.4. Case Management

The dog was diagnosed with an acute hepatopathy of unknown etiology. Differential diagnoses considered included bacterial cholangiohepatitis, acute pancreatitis, previous gallbladder perforation, and neoplasia. Broad-spectrum antibiotic management of ampicillin-sulbactam (30 mg/kg IV q8 h) and enrofloxacin (10 mg/kg IV q24 h) was instituted. N-Acetyl cysteine was administered (140 mg/kg IV once, followed by 70 mg/kg IV q6 h) for antioxidant support.

A nasogastric tube (NGT) was placed to remove gastric residual volume (GRV) due to suspected ileus. Approximately 540 mL fluid was removed in the first 4 hours (20 mL/kg/hr). An IV continuous-rate infusion (CRI) of metoclopramide was administered (0.08 mg/kg/hr), and GRV declined. Antiemetic therapy was continued (ondansetron 0.3 mg/kg IV q8 h), and analgesia was provided (fentanyl 2 *μ*g/kg bolus IV, followed by CRI of 3 *μ*g/kg/hr). Due to the dog's unwillingness to ambulate or urinate, a Foley urinary catheter was placed. Urine output monitoring revealed polyuria (2.7-3.8 mL/kg/hr).

On day 2 of hospitalization, the dog was stable; however, mentation remained abnormal. Vital parameters (rectal temperature, heart rate, and respiratory rate) and blood pressure remained normal. There was minimal GRV present. A low-fat veterinary therapeutic liquid enteral diet was provided via the NGT at a CRI to provide approximately 25% resting energy requirement (RER). Serial monitoring via blood gas analysis (NOVA) showed elevations in BUN and creatinine indicative of an IRIS Stage II acute kidney injury (Table 2).

### 2.5. Additional Diagnostics: Thyroid Panel

**Figure 2:**
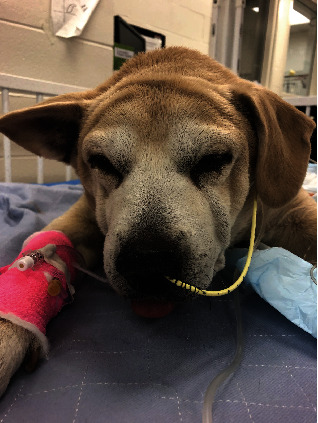
The dog in the ICU with a nasogastric tube (NGT) in place. Note the “tragic” facial expression.

Given concern for possible myxedema coma, including hyperlipidemia, dermatologic signs consistent with an endocrinopathy, abnormal mentation, and “tragic” facial expression (Figure 2), a thyroid panel was submitted to the Michigan State University Veterinary Diagnostic Laboratory (Table 3). Pending these results, there was a plan to initiate intravenous levothyroxine. The panel was consistent with hypothyroidism; however, these results were received after the dog was deceased.

### 2.6. Case Outcome

The patient had multiple instances of inappropriate vocalization overnight and throughout his second day of treatment in the hospital. These vocalizations occurred both while receiving fentanyl CRI (2 mcg/kg/hr) and while not.

The patient became agonal in the evening of his second day in the hospital. Cardiopulmonary resuscitation was initiated, and the patient was intubated. Naloxone and epinephrine were administered IV. Resuscitation efforts were unsuccessful, and the owner elected for humane euthanasia.

### 2.7. Pathology

**Figure 3:**
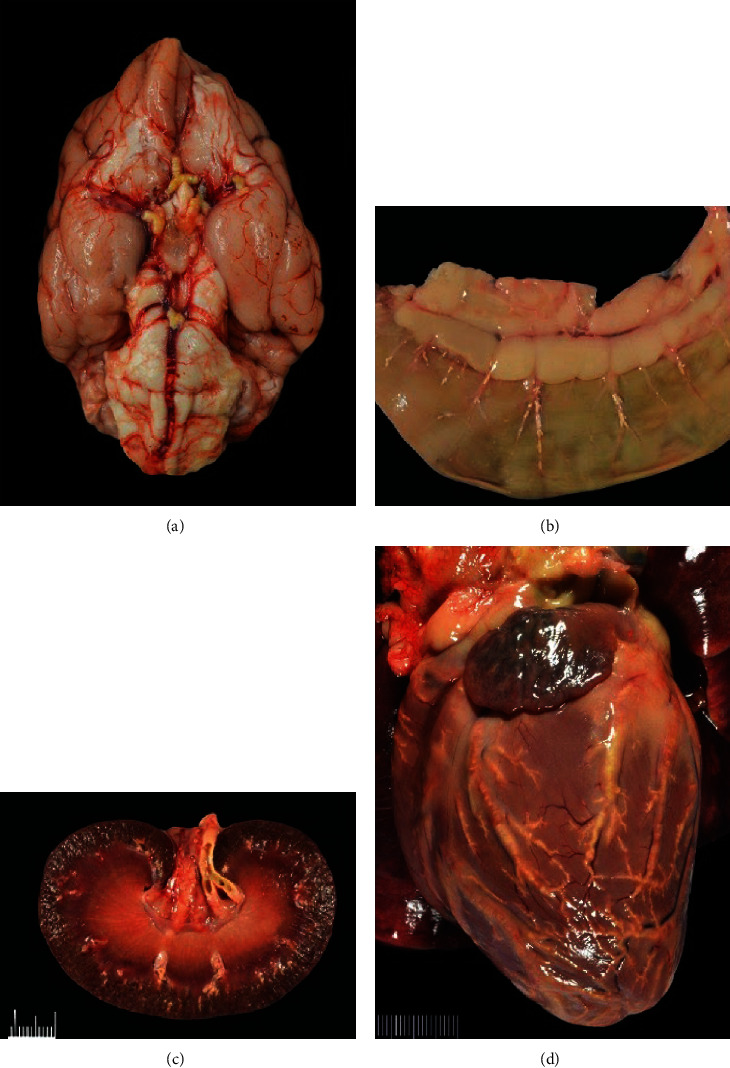
Marked atherosclerotic changes of multiple organs including the (a) brain, (b) gastrointestinal tract, (c) kidney, and (d) heart.

At postmortem examination, there was marked icterus of the abdomen, pinna, and mucous membranes; generalized hypotrichosis; and diffuse subcutaneous edema that most significantly affected the face. There was widespread atherosclerosis, grossly apparent in multiple organ systems, including the gastrointestinal tract, kidneys, heart, testes, and brain; affected arteries were firm and tortuous, thickened by friable but cohesive, yellow-tan material (Figure 3). The thyroid glands were severely atrophied bilaterally. The ventral gallbladder was focally effaced and replaced by fibrinous material that formed a semifirm adhesion with the parietal peritoneum of the cranioventral body wall, consistent with subacute cholecystic rupture and secondary fibrinous peritonitis. The gallbladder was moderately distended by dark brown-black mucoid material, and the wall was thickened and edematous with pinpoint, black foci of hemorrhage and necrosis multifocally across the mucosa. The liver was grossly slightly enlarged with widespread miliary yellow-tan foci of necrosis.

**Figure 4:**
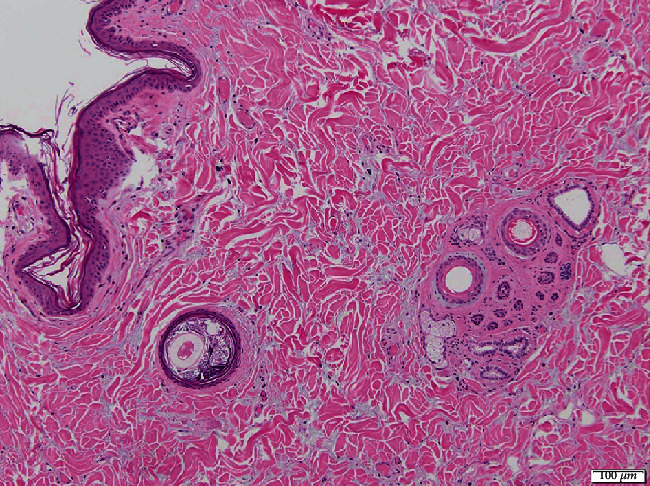
Histopathology of haired skin (hematoxylin and eosin stain, 10x). There is epidermal and adnexal atrophy. Dermal collagen layers are separated by white space (edema) with intervening amorphous amphophilic to basophilic material (mucinosis), changes supportive of cutaneous myxedema.

**Figure 5:**
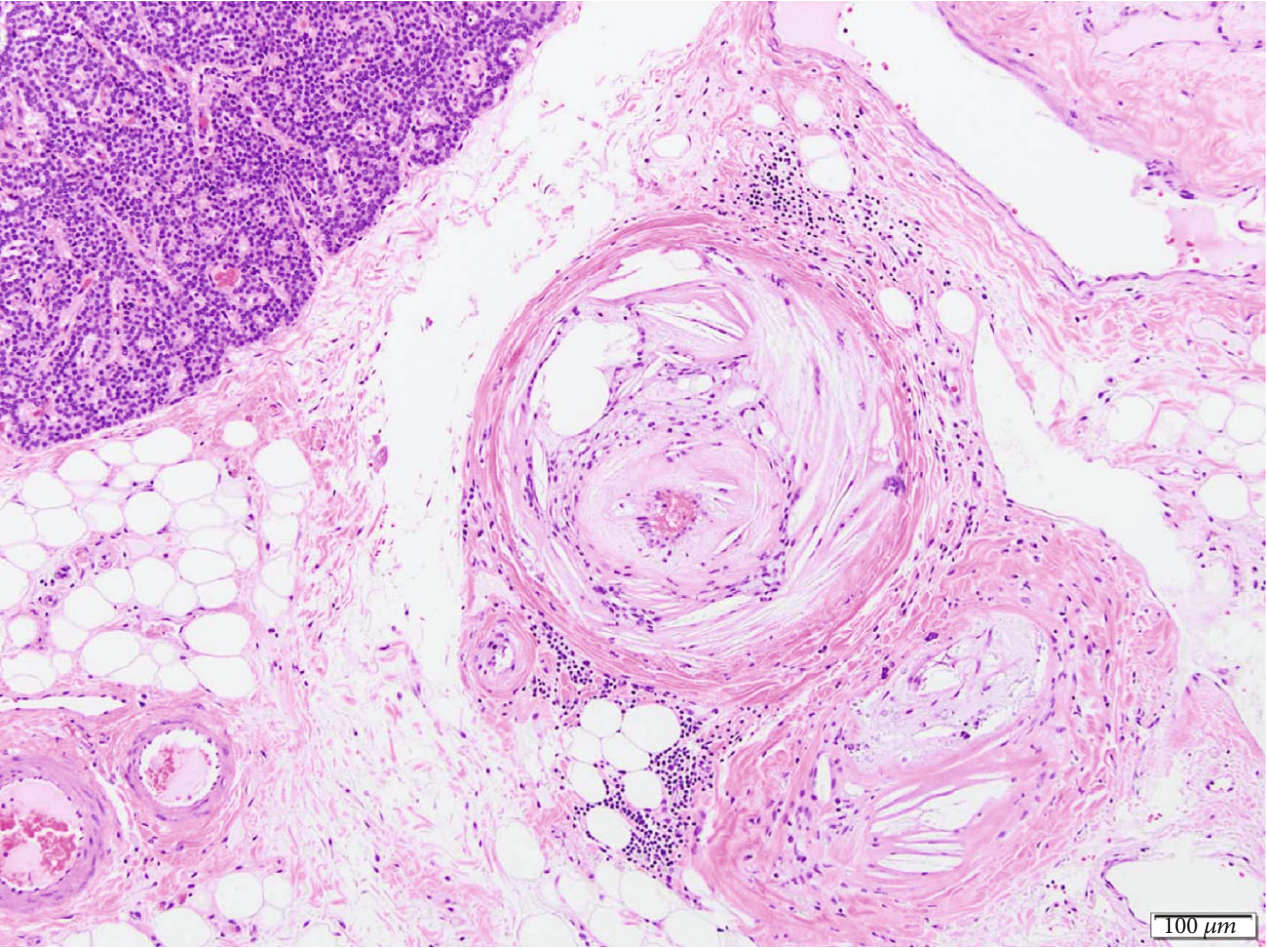
Histopathology of the right thyroid gland (hematoxylin and eosin stain, 10x). Histologically normal parathyroid tissue is seen in the top left. No thyroid follicular epithelium is noted (severe atrophy/loss). The tunica media of this artery is markedly expanded by foam cells, cholesterol clefts, and eosinophilic proteinaceous debris. The tunica adventitia is expanded by fibrous connective tissue, and there is mild lymphoplasmacytic infiltration within the vessel wall and extending into the adjacent fibrofatty tissue.

**Figure 6:**
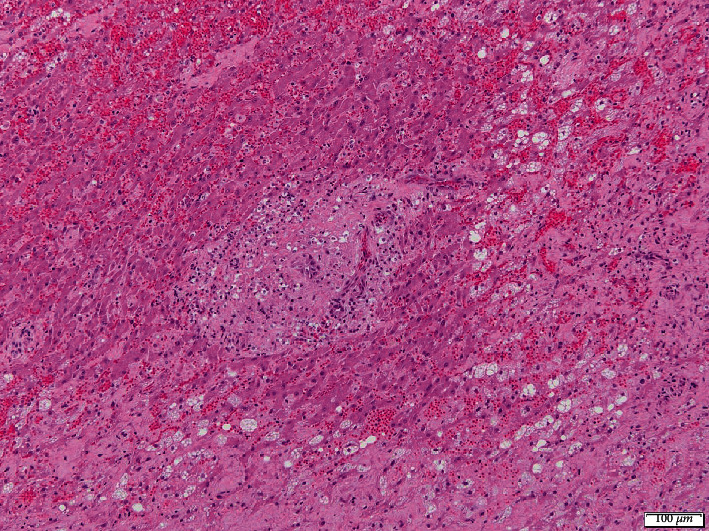
Histopathology of the liver (hematoxylin and eosin stain, 10x). There is complete loss of hepatic cord architecture with widespread biliary and periportal hepatocellular necrosis. Multifocally, hepatocytes are expanded by cytoplasmic vacuolation (hepatocellular lipidosis and/or glycogenosis).

Histologic examination of the skin revealed marked epidermal and adnexal atrophy; superficial dermal collagen fibers were separated by increased clear space containing scant amorphous amphophilic material, supportive of edema and mucinosis, respectively (Figure 4). In most tissues examined, including the cerebrocortical meninges, the arterial and arteriolar lumens were reduced to slit-like openings by circumferential, subintimal expansion with eosinophilic, vacuolated lipid, and cholesterol-laden debris, consistent with marked atherosclerosis. Adventitial and surrounding connective tissues often contained aggregates of lymphocytes and plasma cells. Histological investigations of cerebral tissue did not identify additional changes to account for neurologic deficits. Adjacent to the parathyroid glands, there was a complete paucity of thyroid epithelium/follicles (severe atrophy) with regionally extensive fibrofatty replacement and patchy lymphoplasmacytic infiltrates within the intervening connective tissue (Figure 5). Hepatic changes included widespread necrotizing cholangiohepatitis, cholestasis, and hepatocellular lipidosis (Figure 6).

## 3. Discussion

The patient presented with signs of acute hepatopathy (mixed hepatocellular-cholestatic pattern) characterized at postmortem examination as necrotizing portal hepatitis, fibrinosuppurative peritonitis, and suspected cholecystic rupture. The widespread acute necrotizing cholangiohepatitis was most likely attributable to cholecystic rupture and associated peritonitis [5].

Other significant pathologic findings included widespread atherosclerosis and severe thyroid atrophy, supportive of severely unregulated hypothyroidism; the cutaneous changes were suggestive of myxedema. Based on these findings, we hypothesize that uncontrolled hypothyroidism resulting in atherosclerosis and concurrent hepatobiliary disease together ultimately culminated in myxedema coma (crisis). The presence of these two coexisting pathological processes resulted in a clinical presentation not entirely consistent with either individual condition (e.g., tachypnea, likely resultant from peritonitis associated visceral pain, rather than bradypnea expected with a myxedema crisis). The clinical presentation of the patient and the dual pathological processes thereby complicated disease diagnosis and management.

Based on the absence of microscopic changes in the cerebral tissue, hypothyroidism and atherosclerosis were considered to result in the neurologic signs. Atherosclerosis is the most common cause of arteriosclerosis in humans, but it occurs less frequently in dogs and is characterized mostly by lipid deposition in the tunica media rather than in the tunica intima [6–8]. In humans, development of clinical symptoms associated with atherosclerosis is often attributed to occlusion of the vascular space leading to local ischemia or infarct events [9]. Hypothyroidism has been cited as a risk factor for the development of canine atherosclerosis, likely due to hypercholesterolemia and/or hyperlipidemia associated with this disease [10, 11]. Clinical manifestations of hypothyroid-associated atherosclerosis have been described in dogs in several case reports/series, including a two-year-old male castrated Australian shepherd presenting with cranial nerve and proprioceptive deficits, nystagmus, and vestibular ataxia; four hyperlipidemic Labradors presenting for varying degrees of paresis and ataxia; and a six-year-old Doberman Pinscher presenting for seizures, circling, and a head tilt [12–14]. In one of these patients, necropsy revealed an atrophic thyroid gland and widespread atherosclerosis primarily affecting the cerebral arteries and arteries of the cervical spine [13]. Similar atherosclerotic lesions were identified in our case but were more widespread affecting myocardial, renal, and gastrointestinal vasculature along with the cerebral arteries.

Although the aforementioned case reports highlight the association between hypothyroidism and neurologic deficits, the physiologic pathway for these deficits is unclear. The association between hypothyroidism, hyperlipidemia, and atherosclerosis is well-documented in dogs, and it is hypothesized that aberrant lipid metabolism and deposition may be linked to neurologic deficits in some patients [12, 14]. It has been proposed that hyperlipidemia increases plasma viscosity thus decreasing cortical perfusion [15]. Although we cannot rule out other hypothyroidism-associated pathways for neurologic deficits, in this case, the clinical neurologic deficits (tetraparetic and conscious proprioceptive deficits in all limbs) may have occurred in association with marked CNS atherosclerosis. A proposed mechanism is that atherosclerotic plaques may act as emboli, leading to cerebrocortical ischemia and necrosis [12, 14].

Aside from occasionally mildly elevated liver enzymes (ALP, ALT), a specific association between hypothyroidism and clinically apparent hepatopathy has not been extensively described in the veterinary literature; however, an association between hypothyroidism and gallbladder mucoceles is well described in dogs [16, 17]. Several case reports in human medicine highlight an association between hypothyroidism, hepatic disease, and hyperammonemic coma, with at least one report of acute hypothyroid-induced mixed hepatopathy [18–20]. Cage-side measurement of ammonia is less commonly performed in veterinary medicine, and thus, the ammonia status of the presented case is unknown. However, the presence of concurrent uncontrolled hypothyroidism and acute hepatopathy in this patient may represent an atypical presentation of myxedema coma (crisis) not previously described in veterinary medicine. While it is possible that the hypothyroidism contributed to the hepatopathy in this dog, it is more likely that the cholecystic rupture with associated peritonitis and acute hepatobiliary necrosis precipitated this dog's crisis.

Myxedema coma (crisis) is a rare variant of severe uncontrolled hypothyroidism that has a mortality rate as high as 25-60% in humans [21]. The diagnosis of myxedema coma is classically reliant on clinical signs rather than histological findings [22]. However, myxedema can occasionally be confirmed on histology by presence of subepidermal mucinosis on hematoxylin and eosin staining and confirmed by special stains, such as Alcian blue or periodic acid Schiff [23]. It is thought that a myxedema crisis is due to low intracellular T3 secondary to hypothyroidism, which ultimately leads to a multitude of cellular changes that cause dyshomeostasis [24]. These changes include diminished sensitivity to hypoxia and hypercapnia, altered vascular permeability, water retention, depressed cardiac function, primary or secondary adrenal insufficiency, and decreased gluconeogenesis [24].

In human literature, the leading precipitating events to myxedema crises are infection and septicemia [25, 26]. Other known factors include gastrointestinal bleeding, congestive heart failure, and various drugs. Due to the rarity of myxedema crisis in veterinary medicine, there are currently no studies that demonstrate a similar precipitating causality in canine species. One case study demonstrated that myxedema coma was possibly precipitated in a dog due to beginning diuretic therapy [27]. In this case, a possible contributing factor was cholecystic rupture with associated peritonitis and acute hepatobiliary necrosis.

Treatment of myxedema coma involves establishing an airway and maintaining adequate ventilation, initiating intravenous fluid and levothyroxine therapy, and passive warming to manage hypothermia [28]. The presented patient went agonal and was humanely euthanized prior to the initiation of intravenous levothyroxine therapy.

## Figures and Tables

**Table 1:** Serial CBC results.

Variable	Day 1	Day 2	Reference interval
Hematocrit	33	27	40-59%
MCV	57	68	62-77 FL
MCHC	38.8	36.2	33-36.1 g/dL
RBC morphology	Target cells	Target cells	
Total leukocytes	23.7	17.8	4.8 − 13.9 × 10^9^/L
Segmented neutrophils	20.6	16	2.6 − 10.8 × 10^9^/L
Band neutrophils	2.6	0.4	0 − 0.1 × 10^9^/L
Lymphocytes	0.2	0.5	1 − 4.6 × 10^9^/L
Monocytes	0.2	0.9	0.1 − 1.1 × 10^9^/L
WBC morphology	Döhle bodies	Döhle bodies, reactive lymphocytes	
Platelets	311	259	145 − 463 × 10^9^/L

**Table 2:** Serial biochemistry results. Variables denoted with an asterisk were from blood gas analyzer.

Variable	Day 1	Day 2 7:45 AM	Day 2 9:45 AM	Day 2 4:00 PM	Reference interval
BUN^∗^	21	28		33	9-33 mg/dL
Creatinine^∗^	1.5	1.4		1.9	0.7-1.8 mg/dL
Phosphorus	6.3				2.2-6.3 mg/dL
Total calcium	10.8				9.3-11.3 mg/dL
Sodium^∗^	138.4	144	144.1	145.9	143-150 mmol/L
Potassium^∗^	3.97	2.86	3.47	3.8	3.5-4.8 mmol/L
Chloride^∗^	106.4	104.8	115.8	109.3	111-119 mmol/L
pH^∗^	7.296	7.495	7.378	7.508	7.38-7.48
Bicarbonate^∗^	16.8	28	14	26.5	16.9-24.2 mmol/L
Ionized calcium^∗^	5.6	5.5	5.5	5.5	4.9-5.8 mg/dL
Magnesium^∗^	1.1	1.2	1.0	1.3	1.11-1.53 mg/dL
ALT	4,563				18-108 IU/L
AST	1,585				16-51 IU/L
ALP	2,341				12-133 IU/L
CK	449				53-372 IU/L
Cholesterol	1,272				122-345 mg/dL
Triglycerides	1,118				34-265 mg/dL
Total bilirubin	4.24				0.05-0.2 mg/dL
Albumin	3.1				3.3-4.2 g/dL
Globulin	4.1				1.7-3.4 g/dL
Glucose^∗^	88	147	107	112	78-126 mg/dL
Lactate^∗^	5.5	1.3	3.1	3.8	0.5-3.5 mmol/L

**Table 3:** Thyroid panel results.

Variable	Result	Reference interval
Total thyroxine (TT4)	0	9-45 nmol/L
Total triiodothyronine (TT3)	0.4	0.8-2.1 nmol/L
Free T4	9	6-42 pmol/L
T4 autoantibody	10	0-20%
T3 autoantibody	11	0-10%
TSH	0.97	0-0.58 ng/mL
Thyroglobulin autoantibody	11	0-35%
